# Serum Soluble Receptor for AGE (sRAGE) Levels Are Associated With Unhealthy Lifestyle and Nonalcoholic Fatty Liver Disease

**DOI:** 10.14309/ctg.0000000000000040

**Published:** 2019-05-10

**Authors:** Dana Ivancovsky-Wajcman, Shira Zelber-Sagi, Naomi Fliss Isakov, Muriel Webb, Meir Zemel, Oren Shibolet, Revital Kariv

**Affiliations:** 1 School of Public Health, University of Haifa, Haifa, Israel;; 2 Department of Gastroenterology, Tel-Aviv Medical Center, Tel-Aviv, Israel;; 3 The Sackler Faculty of Medicine, Tel-Aviv University, Tel-Aviv, Israel;; 4 Department of Surgery, Tel-Aviv Medical Center, Tel-Aviv, Israel.

## Abstract

Nonalcoholic fatty liver disease (NAFLD) has been demonstrated to be positively associated with serum advanced glycation end products (AGEs) and negatively with soluble receptor for AGE (sRAGE) in a few small studies. We aimed to test the association between lifestyle and sRAGE levels and the association between sRAGE levels or AGEs intake and NAFLD, insulin resistance (IR), and elevated alanine aminotransferase (ALT).

**METHODS:**

Cross-sectional analysis among participants of a screening study. Fasting blood tests and serum sRAGE levels were obtained. NAFLD and insulin resistance were evaluated by ultrasonography and homeostasis model assessment, respectively. Nutritional intake was measured by food frequency questionnaire, and the intake of dietary AGEs was calculated.

**RESULTS:**

A total of 743 subjects were included (52.6% men, mean age 58.83 ± 6.58 years, 38.7% NAFLD). Exercise was independently protective from low sRAGE levels (odds ratio [OR] = 0.71, 95% confidence interval 0.52–0.97, *P* = 0.031). Pack-years, working time, and sedentary time (OR = 1.51, 1.03–2.22, *P* = 0.036; OR = 1.66, 1.18–2.35, *P* = 0.004; OR = 1.64, 1.18–2.29, *P* = 0.004, respectively), and intake of red and/or processed meat or processed meat alone (OR = 1.01, 1.04–2.21, *P* = 0.045; OR = 1.49, 1.00–2.21, *P* = 0.048, respectively) were associated with increased odds for low sRAGE levels. Low sRAGE levels were independently associated with elevated ALT (OR = 1.69, 1.11–2.57, *P* = 0.014) and NAFLD with elevated ALT (OR = 2.17, 1.23–3.83, *P* = 0.007). High intake of dietary AGEs was associated with IR (OR = 2.04, 1.25–3.34 *P* = 0.004).

**DISCUSSION:**

Lifestyle is associated with sRAGE levels and, in turn, low levels of sRAGE are associated with NAFLD and elevated ALT.

## INTRODUCTION

Nonalcoholic fatty liver disease (NAFLD), the hepatic manifestation of insulin resistance (IR), is one of the most important causes of liver disease worldwide (1) and is the most common cause of abnormal alanine aminotransferase (ALT) (2). Unhealthy Western lifestyle, which is characterized by smoking (3,4), poor diet, long sedentary and working time, and low performance of exercise (*triple hit behavioral phenotype*), has a well-established association with NAFLD and liver damage (5,6). Specifically, dietary patterns that are rich in red and/or processed meat (7) and sugar-sweetened beverages (8) are significant in NAFLD etiology (9–11). The mechanisms by which diet and other lifestyle factors lead to NAFLD or its progression are not completely understood. In recent years, the role of advanced glycation end products (AGEs)/receptor for AGEs (RAGE) axis has been studied in relation to NAFLD. AGEs are the end products of the Maillard reaction, a nonenzymatic reaction between reducing sugars and the free amino groups of proteins (12–14). Although AGEs are normally formed endogenously, exogenous sources appear to also increase serum AGEs levels, including tobacco use and intake of high heat-cooked food like processed food, especially meat (12,13,15). An interaction between AGEs and its cell-surface receptor RAGE stimulates activation of nuclear factor kB, thrombogenesis, vascular inflammation, pathological angiogenesis (12,13), and oxidative stress (16). There are several isoforms of soluble RAGE (sRAGE), the main 2 are sRAGE and endogenous secretory RAGE (esRAGE). The first is derived from cell surface cleavage mechanisms and the second is derived from pre-mRNA alternative splicing (17).

In contrast to RAGE, sRAGE lacks the intracellular domain (18), prevents the binding of extracellular AGEs to the cell-surface RAGE, and inhibits the AGE/RAGE signaling (17). Thus, sRAGE exerts protective effects in pathologic, metabolic, and inflammatory conditions (17). Indeed, few studies demonstrated a protective association between high sRAGE levels and liver damage including NAFLD (19–21), nonalcoholic steatohepatitis (NASH) (20), and liver cancer (22). In addition, low serum sRAGE levels were found to be positively associated with elevated ALT and IR (23). Although the protective role of sRAGE is increasingly explored, there is uncertainty about the factors related with serum sRAGE levels. An inverse association between sRAGE levels and anthropometric measures, weight (24), body mass index (BMI) (24–26), and waist circumference (24), has been demonstrated. However, the possible effect of lifestyle on sRAGE levels is less studied. Exercise was positively correlated with elevated sRAGE levels in both observational (27) and clinical (18,19) studies. In addition, smoking and pack-years were demonstrated to be associated with lower sRAGE levels in a cross-sectional study (28). However, there is lack of data on the association between nutrition in general and meat consumption in particular, and serum sRAGE levels. The current study aimed to test the association between lifestyle habits and sRAGE levels and, in turn, the association between sRAGE or AGEs levels and NAFLD, IR, and elevated ALT. We hypnotize that unhealthy lifestyle habits will be negatively associated with sRAGE levels, and, in turn, low sRAGE levels will be positively associated with NAFLD, IR, and elevated ALT.

## METHODS

### Study design and population

This was a cross-sectional study among consecutive 40- to 70-year-old subjects who underwent screening colonoscopy at the Department of Gastroenterology and Hepatology in the Tel-Aviv Medical Center and agreed to participate in a metabolic and hepatic screening study between the years 2010 and 2015 (convenience sample) (previously described in detail (7)). Exclusion criteria included presence of Hepatitis B virus surface antigen (HBsAg) or anti-hepatitis C virus (HCV) antibodies, fatty liver suspected to be secondary to hepatotoxic drugs, inflammatory bowel disease, celiac disease, and excessive alcohol consumption (≥30 g/d in men or ≥20 g/d in women) (29,30). In addition, subjects who reported an unreasonable caloric intake were excluded: outside the acceptable range for men 800–4,000 kcal/d and for women 500–3,500 kcal/d (31).

The study was approved by the Tel-Aviv Medical Center Institutional Review Board, and all patients signed an informed consent. A graphical explanation of the study hypothesis is available as an Addendum (see Supplementary Digital Content 1, http://links.lww.com/CTG/A47).

### Data collection

Study participants were invited for a single day visit, in which they underwent fasting blood tests, liver ultrasound, interview using a structured questionnaire, assembled by the Israeli Ministry of Health (32), including demographic details and lifestyle. In addition, they completed food frequency questionnaire (FFQ).

Fatty liver was diagnosed by abdominal ultrasonography (AUS) using standardized criteria and performed in all subjects with the same equipment (EUB-8500 scanner Hitachi Medical Corporation, Tokyo, Japan) and by the same experienced radiologist (Webb M) as previously described (8). The ratio between the brightness level of the liver and the right kidney cortex was calculated to determine the hepatorenal index, which has been previously validated against liver biopsy (33).

IR was evaluated by high homeostasis model assessment (HOMA) score, defined by the 75th percentile (upper quartile, Q4) of the study sample HOMA levels, as accepted (34) (corresponding to a value > 3.31 for the entire sample and >4.68 for subjects with NAFLD).

Type 2 diabetes was defined as fasting glucose ≥126 mg/dL and/or HbA1C ≥ 6.5% and/or use of diabetic medications (35). Since in diabetes serum insulin concentrations may start to decline (36), the patients with diabetes who had no IR according to the upper quartile of HOMA levels (n = 44) were considered as having IR. Abdominal obesity was defined as waist circumference ≥102 for men and ≥88 for women (37).

Elevated ALT was defined according to the American College of Gastroenterology clinical guidelines: ALT> 33 IU/L for men and ALT> 25 IU/L for women (38). Serum sRAGE levels (all isoforms) were measured by human RAGE Quantikine ELISA kit (R&D Systems, MN) in frozen sera kept at −80 °C. Low sRAGE was defined as sRAGE levels below the cutoff of the population's first tertile (<1,013 pg/mL).

### Nutritional variable evaluation and definitions

The semiquantitative FFQ, which was assembled by the Food and Nutrition Administration, Ministry of Health and tailored to the Israeli population, is composed of 117 food items with specified serving sizes. The nutrient components of each food item were taken from the Israeli National Nutrient Database, Food and Nutrition Administration, Ministry of Health. Meat types were categorized as accepted to “red”, “white” (chicken), and processed meat (39,40) as was previously described in detail (7). *Processed meat* was defined as meat that has been transformed through salting, smoking, or other processes to enhance flavor or improve preservation (40). In addition, estimation of dietary AGEs consumption was calculated based on food items from the FFQ using specific database (16) that has been used in several other studies (41,42). In general, the calculation relates to the following food groups: red meat, poultry, fish, bread and cereals, dairy products, and oils. For each food item, we calculated the amount of AGEs (AGEs content per 100 gr food item obtained from the database multiplied by the food item weight) and then summed total AGEs from all foods.

### Lifestyle variable evaluation and definitions

The exercise questionnaire included a detailed list of aerobic activities and resistance training.

Pack-years was defined as cigarettes per day divided to 20 cigarettes per pack, multiplied by years of smoking. In addition, the lifestyle questionnaire included questions about the number of hours spent sedentary, screen time (computer or television) or reading. Physical effort during work was defined as “easy” (sedentary work or mostly standing) or “strenuous” (walking or having physical effort during work). Unemployed subjects were excluded (n = 237) from this analysis. Data about potential confounders such as anthropometrics, gender, age and caloric intake were collected.

### Statistical analysis

Statistical analyses were performed using SPSS version 23 (IBM-SPSS Armonk, NY) software. Continuous variables are presented as means ± SD. To test differences in continuous variables between 2 or 3 groups, the independent sample *t* test and ANOVA were performed, respectively. Associations between nominal variables were performed with the Pearson χ^2^ test. In both χ^2^ test and ANOVA, *P* for trend was calculated. A multivariate logistic regression analysis was performed to test the adjusted association between sRAGE and nutritional intake and NAFLD or high ALT adjusting for potential confounders (relevant variables that were different between low and high sRAGE levels as presented in Table 1). Odds ratio (OR) and 95% confidence interval are presented. *P* value of <0.05 was considered statistically significant for all analyses.

**Table 1.**
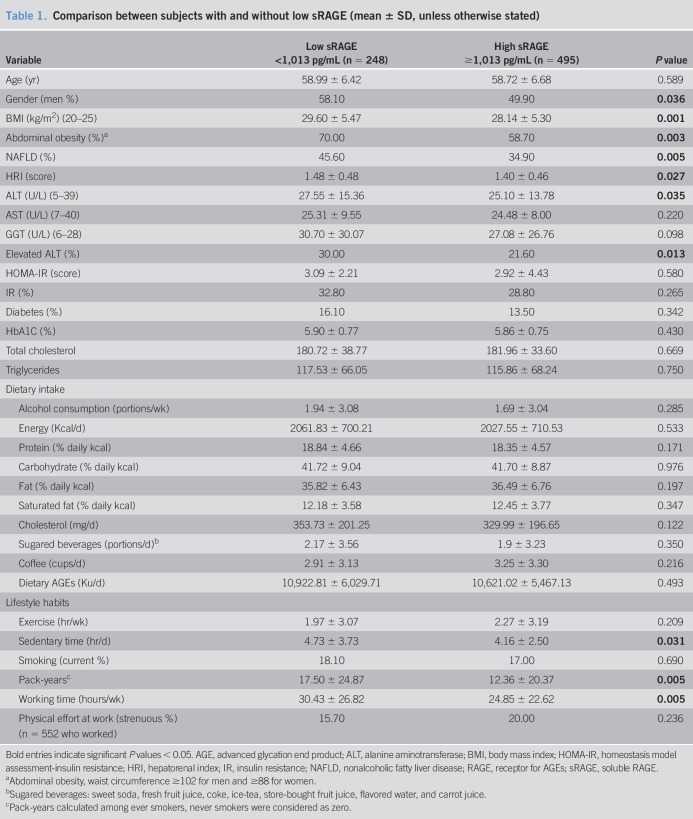
Comparison between subjects with and without low sRAGE (mean ± SD, unless otherwise stated)

## RESULTS

Description of the study population and comparison between subjects with high and low (by first tertile) sRAGE levels is provided in Table 1.

**Figure 1.**
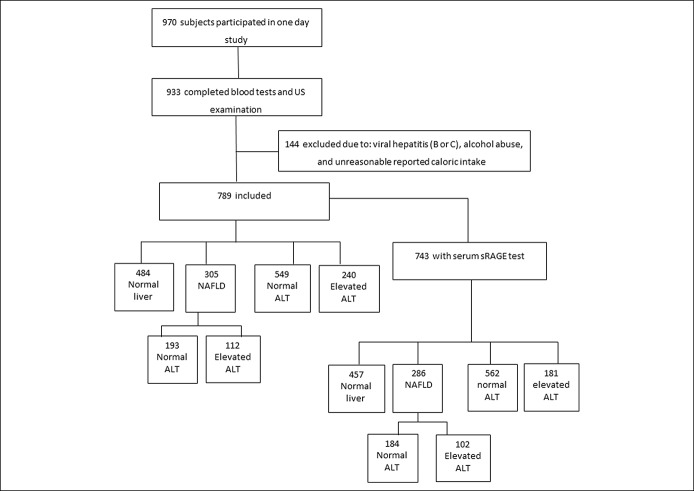
Flow chart of the study population.

Out of 970 subjects who participated in the study, 789 subjects were eligible, had all data, and were included in the analysis, as previously described (7). Of those, 743 subjects had serum sRAGE levels analysis (flow chart of the study population appears in Figure 1).

In the final sample of 789 subjects, 52.6% were men, mean age was 58.83 ± 6.58 years, and mean BMI was 28.54 ± 5.43 kg/m^2^. NAFLD was diagnosed in 38.7% (n = 305) of participants and elevated ALT was observed in 24.9% (n = 196) (2 subjects with missing data), whereas 14.2% (n = 112) had both NAFLD and elevated ALT. IR was diagnosed in 30.4% (n = 240) of participants (3 subjects with missing data) and type 2 diabetes among 14.8% (n = 117). The subsample of 743 subjects had similar characteristics in all parameters (52.6% men, mean age 58.81 ± 6.59 years).

Subjects with low sRAGE levels had higher prevalence of men, higher mean BMI, and higher prevalence of abdominal obesity, and these variables were considered as potential confounders. In addition, the prevalence of NAFLD was higher among subjects with low sRAGE levels, supported by higher hepatorenal index, as well as a greater prevalence of elevated ALT. Subjects with low sRAGE had longer smoking pack-years, sedentary time, and working hours.

Multivariate analysis of the association between lifestyle parameters and low sRAGE levels is provided in Table 2.

**Table 2.**
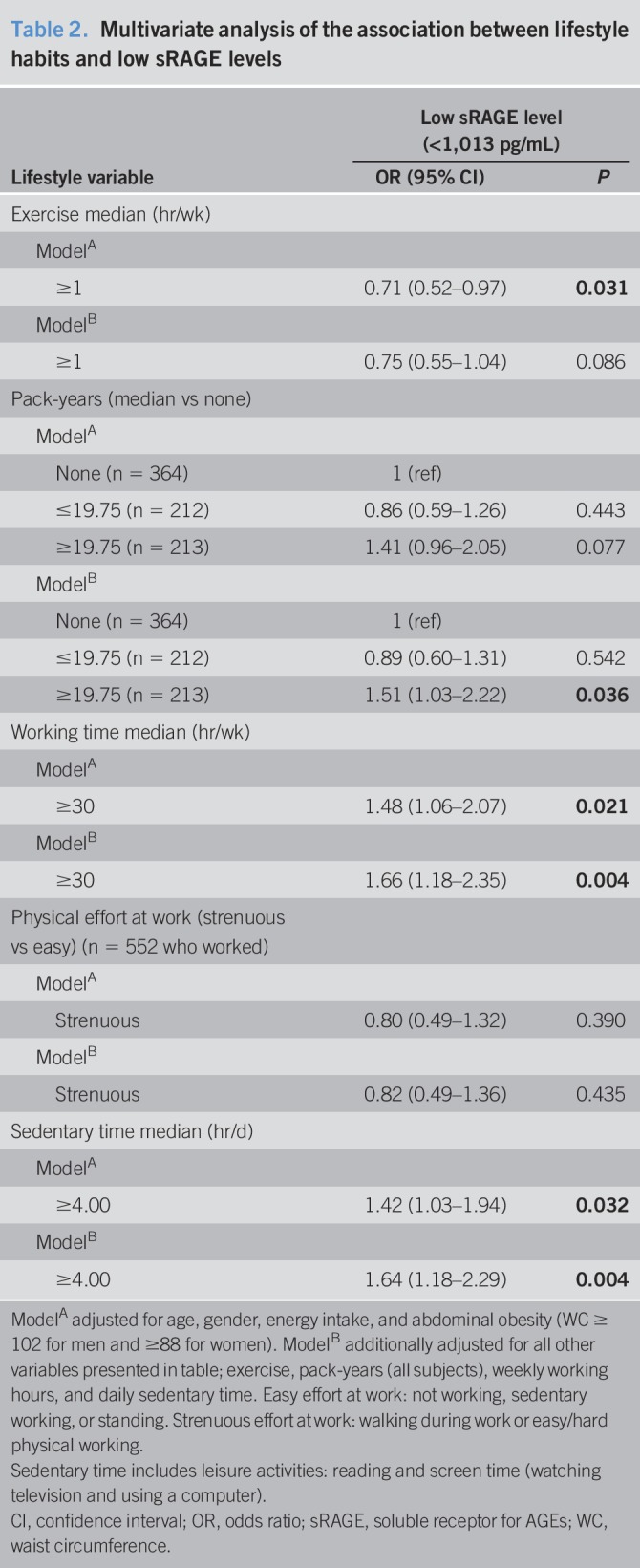
Multivariate analysis of the association between lifestyle habits and low sRAGE levels

High level of exercise was independently protective from low sRAGE levels (OR = 0.71, 95% confidence interval 0.52–0.97, *P* = 0.031) adjusting for age, gender, caloric intake, and abdominal obesity (Table 2, model A), but not with further adjustment for other lifestyle habits. In contrast, longer pack-years, working time, and sedentary hours (above median) were associated with increased odds for low sRAGE, adjusting for potential confounders and lifestyle habits (OR = 1.51, 1.03–2.22, *P* = 0.036; OR = 1.66, 1.18–2.35, *P* = 0.004; OR = 1.64, 1.18–2.29, *P* = 0.004, respectively) (Table 2, fully adjusted model B)*.*

**Figure 2.**
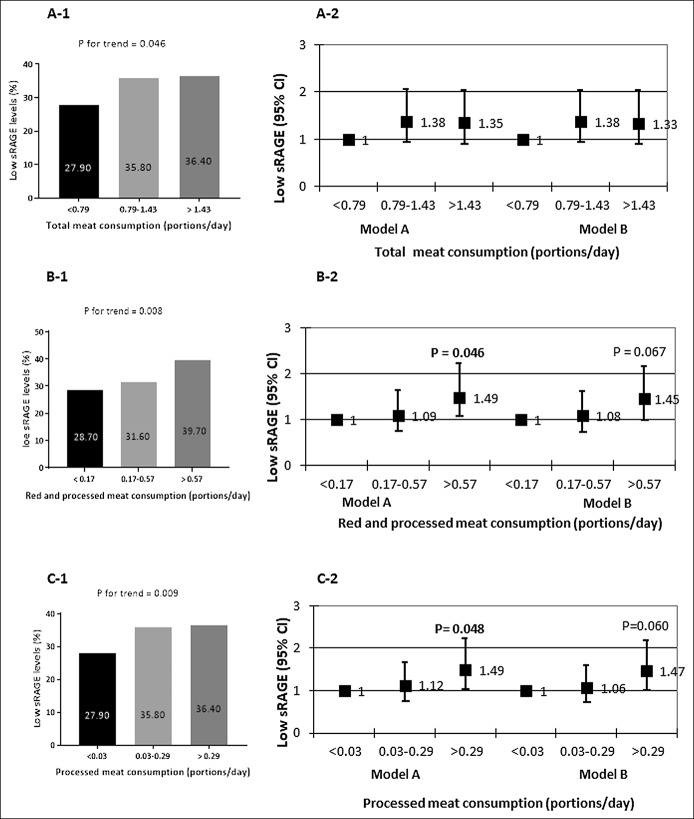
Dose-response univariate and multivariate association between tertiles of total meat (**a1 and a2**), red and/or processed meat (**b1 and b2**), processed meat consumption (**c1 and c2**), and low sRAGE. Univariate *P* values are calculated by χ^2^ test. Multivariate OR model A adjusted for age, gender, energy intake (Kcal), and abdominal obesity (WC ≥ 102 cm for men and ≥88 cm for women); Model B additionally adjusted for working time (hr/d) and pack-years (N in each tertile; 248, 248, 247, respectively). sRAGE, soluble receptor for advanced glycation end product; OR, odds ratio; WC, waist circumference.

Univariate and multivariate analyses of the association between meat consumption and low sRAGE levels are shown in Figure 2.

The prevalence of low sRAGE levels was significantly higher across the increased consumption of total, red, and/or processed meat, in a dose-response manner (Figure 2, a-1, b-2, and c-1). In a multivariate analysis, the upper tertiles of red and/or processed meat or processed meat intake alone were associated with increased odds for low sRAGE levels (OR = 1.01, 1.04–2.21, *P* = 0.045; OR = 1.49, 1.00–2.21, *P* = 0.048, respectively), adjusting for age, gender, caloric intake, and abdominal obesity (Figure 2, b-2 and c-2, model A**)**, but further adjustment for other lifestyle habits that were associated with meat consumption (pack-years and working time) attenuated the significance of associations. There was no association with total meat consumption.

**Figure 3.**
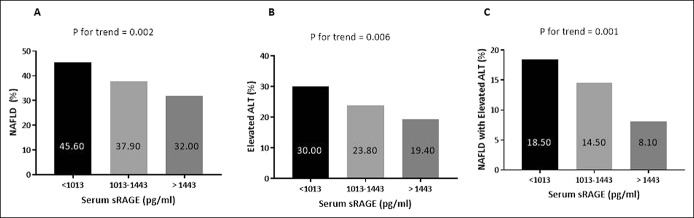
Dose-response univariate association between tertiles of serum sRAGE levels (**a1–a3**) with NAFLD, elevated ALT, or NAFLD with elevated ALT. Univariate *P* values are calculated by χ^2^ test (N in each tertile; 248, 248, 247, respectively). ALT, alanine aminotransferase; NAFLD, nonalcoholic fatty liver; sRAGE, soluble receptor for advanced glycation end product.

Univariate and multivariate analyses of the association between dietary AGEs, sRAGE levels, and NAFLD are shown in Table 3 and Figure 3.

**Table 3.**
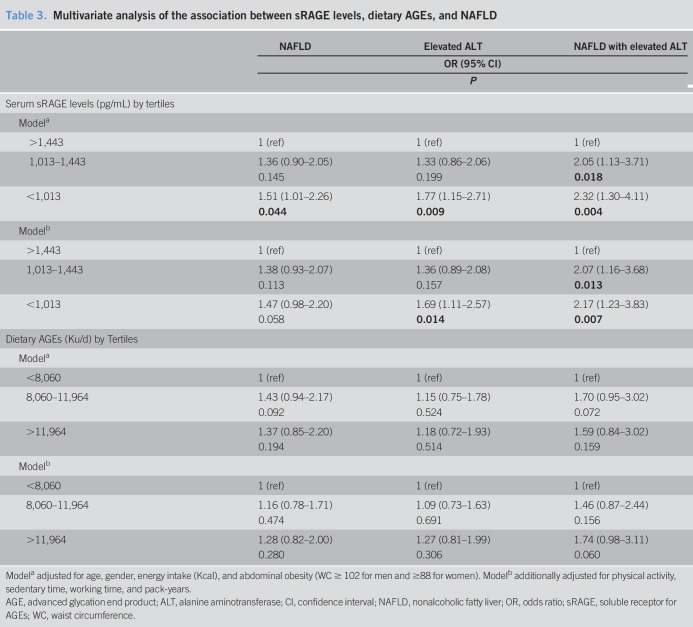
Multivariate analysis of the association between sRAGE levels, dietary AGEs, and NAFLD

The prevalence of NAFLD, elevated ALT, and NAFLD with elevated ALT was lower as sRAGE levels were higher in a dose-response association (Figure 3a–c). In a multivariate analysis, the low serum sRAGE levels (<1,013 pg/mL) were associated with increased odds for NAFLD (OR = 1.51, 1.01–2.26, *P* = 0.044) adjusted for age, gender, caloric intake, and abdominal obesity. In the full model, with further adjustment for all lifestyle habits, which were associated with sRAGE (Table 2), low sRAGE levels were associated with elevated ALT (OR = 1.69, 1.11–2.57, *P* = 0.014), and significant dose-response association was demonstrated between serum sRAGE levels and NAFLD with elevated ALT (Table 3, model B).

Exclusion of diabetic subjects did not change any of the above-mentioned associations (data not shown).

**Figure 4.**
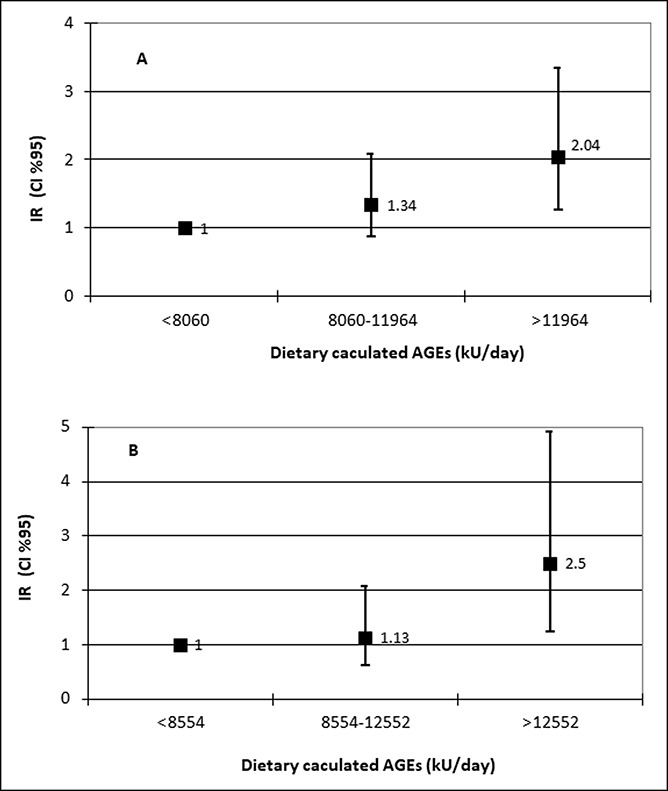
Dose-response multivariate association between tertiles of dietary AGEs and IR among the entire population (**a**) or NAFLD subjects (**b**). Multivariate OR adjusted for age, gender, energy intake (Kcal), abdominal obesity, exercise (hr/d), working time (hr/d), sedentary time (hr/d), and pack-years [N of cases/N in tertile; 62/261, 79/263, 99/262 (**a**) or 48/101, 50/102, 69/102 (**b**) for the first, second and third tertile, respectively]. NAFLD, nonalcoholic fatty liver; AGE, advanced glycation end product; IR, insulin resistance.

Dietary AGEs were not associated with elevated ALT and/or NAFLD. However, the upper tertile of dietary AGEs (>11,964 kU/d) was associated with increased odds for IR in the entire population (OR = 2.04, 1.25–3.34, *P* = 0.004), and among NAFLD subjects (dietary AGEs > 12,552 kU/d) (OR = 2.50, 1.24–4.91, *P* = 0.010), adjusted for age, gender, caloric intake, abdominal obesity, and lifestyle habits as detailed in Table 2 (Figure 4a,b).

## DISCUSSION

There is a wide agreement that unhealthy lifestyle plays a major role in the development and progression of NAFLD (6,11). However, there is still a need for further refinement of the complex interplay between lifestyle risk factors and NAFLD. This study thoroughly analyzed the association between a wide spectrum of diet and other NAFLD-related lifestyle factors, including AGEs intake, and sRAGE as a link to increased risk for NAFLD. Generally, we have shown that unhealthy lifestyle is associated with lower sRAGE levels which in turn is strongly and independently related with NAFLD and elevated ALT in a dose-response manner. Although exercise was positively associated with serum sRAGE levels, smoking burden, sedentary and working time, as well as red and/or processed meat consumption were negatively associated with sRAGE levels, adjusting for other lifestyle habits.

Our results are in accordance with the few studies published on this topic, showing that exercise (18,27) or lifestyle intervention (19) may be effective in increasing serum sRAGE levels (18,27). Similarly, our finding that smoking burden is associated with low sRAGE levels supports previous findings, showing a negative association between pack-years and sRAGE levels among normal weight patients with chronic obstructive pulmonary disease (43) or in the general population (28). In contrast to our finding that longer sedentary and working time are associated with low serum sRAGE levels, in Momma et al. study (44), there was no difference in desk work time between esRAGE tertiles. Our study demonstrated a novel association between red and especially processed meat consumption, and low sRAGE levels in a dose-response manner, which was maintained following adjustment for all other lifestyle risk factors. This association is interesting since the association between meat consumption, especially red and processed meat, and NAFLD was demonstrated in several studies (7,8,45).

The mechanisms linking between lifestyle and sRAGE levels are unknown. Tobacco use, high meat consumption, and processed food consumption alter AGEs body pool and may indirectly affect sRAGE levels (12). Specifically, the association between sRAGE and meat may stem from the fact that processed meat contains, in addition to metabolically unhealthy compounds as sodium (46) and other preservatives (47), a significant amount of AGEs (47,48). Dietary AGEs were associated in our study with increased IR in both the entire population and among NAFLD-diagnosed subjects. This association is supported by clinical trials and meta-analyses indicating beneficial effect of dietary AGEs restriction in the reduction of HOMA-IR (12,42,49) and cardiometabolic risk factors (42). Indeed, an inverse association has been demonstrated between sRAGE levels and IR and the metabolic syndrome components (20,25,50,51). In addition, these unhealthy lifestyle habits may cause inflammation and oxidative stress (52). An inverse correlation has been demonstrated between oxidative stress biomarkers and sRAGE levels (53), suggesting the hypothesis that oxidative stress may mediate the association between unhealthy lifestyle and low sRAGE levels.

Next, our study demonstrated that low serum sRAGE levels are associated with increased odds for NAFLD, elevated ALT, and NAFLD with elevated ALT. Our findings are in agreement with previous cross-sectional and case-control studies showing that low sRAGE levels increase the odds for NAFLD, adjusting for obesity measures (19,23). Elevated ALT was added to the outcomes in our study since it signals liver inflammation and injury and a higher likelihood for NASH (38), all potentially relevant to the proinflammatory and anti-inflammatory effect of AGEs and sRAGE, respectively (17). Interestingly, low sRAGE had a stronger association with elevated ALT by itself or combined with NAFLD compared to NAFLD alone. Similarly, low sRAGE has been demonstrated by previous studies to be associated with elevated ALT (19,20,23) and indeed also with proven NASH (20). These findings are also supported by similar association of high serum AGEs/sRAGE ratio with NAFLD and elevated ALT (23). Altogether, our finding and results from previous studies support the notion that sRAGE may serve as a useful marker and therapeutic target for NAFLD.

The study has several limitations to consider. First, the cross-sectional design of the study does not allow causal inference, and our findings need to be confirmed in prospective studies. Second, lifestyle habits and meat consumption were self-reported and thus prone to a report bias. However, since the participants and the research team were both blinded to the AUS and blood tests results, it is a nondifferential repot bias and therefore it may have only weakened the observed associations. In addition, the external validity of the associations with diet needs to be confirmed among diverse populations with different dietary and lifestyle characteristics. Moreover, the calculated AGEs intake is based on Americans food items that may be different from the Israeli food items. Third, the diagnosis of NAFLD was established by AUS, which is not the gold standard. However, it is the most accepted and common screening method for NAFLD in a general population (29).

In conclusion, exercise is positively associated whereas meat consumption, smoking duration, working time, and sedentary time are negatively associated with serum sRAGE levels. In turn, low levels of sRAGE are associated with both NAFLD and elevated ALT levels. These findings, if confirmed in prospective studies, may help to understand the molecular mediators of lifestyle factors and their role in the pathogenesis of NAFLD and aid in designing future treatment strategies.

## CONFLICTS OF INTEREST

**Article guarantor:** Shira Zelber Sagi, RD, PhD.

**Specific author contributions:** Ivancovsky-Wajcman Dana, RD and Zelber-Sagi Shira, RD, PhD, contributed equally to this work. I.-W.D. designed the study, carried out the data collection and analysis, and wrote the manuscript; Z.-S.S. conceived and designed the study, supervised the data collection and analysis, and wrote the manuscript; F.I.N. designed the study and carried out the data collection; W.M. performed the ultrasonography evaluation and critically reviewed the manuscript; Z.M. carried out the data collection; S.O. critically reviewed the manuscript; and K.R. conceived and designed the study, supervised the data collection, and critically reviewed the manuscript.

**Financial support:** Research Grants and Fellowships Fund on Food and Nutrition and their Implications on Public Health, The Israeli Ministry of Health.

**Potential competing interests:** None.

**Study Highlights**

**WHAT IS KNOWN**

✓ NAFLD is positively associated with AGEs and negatively associated with sRAGE levels.
✓ Smoking is negatively associated with sRAGE levels.

**WHAT IS NEW HERE**

✓ sRAGE levels are negatively and independently associated with elevated ALT and with NAFLD and elevated ALT.
✓ Red and/or processed meat consumption, pack-years, working time, and sedentary time are negatively associated with sRAGE levels.
✓ Exercise is positively associated with sRAGE levels.

**TRANSLATIONAL IMPACT**

✓ sRAGE may serve as a therapeutic target for NAFLD.

## Supplementary Material

SUPPLEMENTARY MATERIAL
