# Supplementary material for: Serum Soluble Receptor for AGE (sRAGE) Levels Are Associated With Unhealthy Lifestyle and Nonalcoholic Fatty Liver Disease
Source: Clin Transl Gastroenterol. 2019 May 10;10(5):e00040. doi: 10.14309/ctg.0000000000000040 (PMC6602762; doi:10.14309/ctg.0000000000000040)
Supplement: SUPPLEMENTARY MATERIAL [file ct9-10-e00040-s001.docx]

**Addendum**

**Table 4. Comparison between subjects diagnosed with NAFLD vs. normal liver**

| **Variable** | **Normal liver (n=484)** | **Fatty liver**  **(n=305)** | **P value** |
| --- | --- | --- | --- |
| Age (years) | 58.79±6.75 | 58.90±6.30 | 0.829 |
| Gender (men %) | 49.80 | 57.00 | **0.047** |
| BMI (kg/m^2^) (20-25) | 26.64±4.56 | 31.56±5.33 | **<0.001** |
| †Abdominal obesity (%) | 46.40 | 86.50 | **<0.001** |
| HRI (score) | 1.12±0.12 | 1.88±0.42 | **<0.001** |
| ALT (U/L) (5-39) | 23.00±11.68 | 30.74±16.40 | **<0.001** |
| AST (U/L) (7-40) | 23.89±7.35 | 26.32±9.95 | **<0.001** |
| GGT (U/L) (6-28) | 24.93±25.58 | 33.52±29.52 | **<0.001** |
| Elevated ALT (%) | 17.40 | 36.80 | **<0.001** |
| HOMA-IR (score) | 2.17±1.37 | 4.28±5.56 | **<0.001** |
| Insulin resistance (%) | 15.20 | 54.80 | **<0.001** |
| Diabetes (%) | 7.40 | 26.60 | **<0.001** |
| HbA1C (%) | 5.73±0.61 | 6.10±0.91 | **<0.001** |
| Total cholesterol | 182.17±33.63 | 180.70±38.47 | 0.584 |
| Triglycerides | 99.00±55.81 | 142.90±73.86 | **<0.001** |
| *Dietary intake* | | | |
| Alcohol consumption (portions/ week) | 1.71±2.86 | 1.75±3.21 | 0.847 |
| Energy (Kcal/day) | 1996.33±694.30 | 2073.17±706.55 | 0.133 |
| Protein (% daily Kcal) | 18.17±4.66 | 19.11±4.55 | **0.005** |
| Carbohydrate (% daily Kcal) | 42.20±8.85 | 40.83±8.92 | **0.035** |
| Fat (% daily Kcal) | 36.13±6.51 | 36.61±6.85 | 0.324 |
| Saturated fat (% daily Kcal) | 12.25±3.76 | 12.58±3.55 | 0.232 |
| Cholesterol (mg/day) | 320.27±177.91 | 358.60±213.8 | **0.007** |
| §Sugared beverages  (portions/day) | 2.11±3.27 | 1.85±3.38 | 0.297 |
| Coffee (cups/day) | 3.15±3.15 | 2.96±3.36 | 0.406 |
| Dietary  AGEs (Ku/day) | 10345.50±5460.01 | 11185.07±5776.06 | **0.040** |
| *Lifestyle habits* | | | |
| Exercise (hours/week) | 2.46±3.43 | 1.71±2.49 | **<0.001** |
| Sedentary time (hours / day) | 4.17±2.88 | 4.55±3.01 | 0.075 |
| Smoking (current %) | 16.50 | 17.40 | 0.757 |
| ⱡPack years | 12.50±20.60 | 16.33±23.73 | **0.020** |
| Working time (hours/ week) | 26.77±24.77 | 27.70±22.86 | 0.599 |
| Physical effort at work  (strenuous %)  (n=552 who worked) | 20.70 | 15.20 | 0.107 |

**Abbreviations:** NAFLD, non-alcoholic fatty liver disease

**Notes**: †Abdominal obesity, waist circumference ≥102 for men and ≥88 for women; §Sugared beverages: sweet soda, fresh fruit juice, coke, ice-tea, store-bought fruit juice, flavored water, carrot juice.

ⱡPack years calculated among ever smokers, never smokers were considered as zero.

**Food Frequency Questionnaire (FFQ)**

Instructions for filling up the FFQ:

In the columns **“A”** and **“B”** the different foods and drinks are listed, with the group name in Column “**A”** and the foods in each grouping in column “**B**”.

-In column **“C”** the serve size is defined (a particular serve size for each food), next to the food item.

-In column **“D”** you are asked to list, next to each item, the number of serves of the listed foods you eat or drink each day.

-If you eat or drink the item several times a week, but not on a daily basis, please mark, in column **“E”**, the number of serves you eat or drink a week.

-If you eat or drink the food item less than once a week, and/or you do not eat it at all, check this in column **“F”**.

Example: The subject eats: milk 1%- once a week, chewing gum 5 pieces a day (every day), drinks 10 cups of Pepsi a week, and all the other items on the list he does not eat at all - the table should be filled out as follows:

| **A** | **B** | **C** | **D** | **E** | **F** |
| --- | --- | --- | --- | --- | --- |
| **Food Group** | **Food item** | **Serve size** | **No. of serves/day** | **No. of serves per week** | **Eats or drinks the item less than once a week, or never** |
| **Dairy products** | Milk 1% | 1 cup |  | 1 |  |
|  | Sour cream | 1 cup |  |  | X |
| **Meat, fish** | Processed schnitzel | 1 serve |  |  | X |
| **Vegetables** | Cabbage salad | 1 cup |  |  | X |
| **Sweets,**  **Snacks** | Chewing gum | 1 piece | 5 |  |  |
| **Drinks** | Cola | 1 cup |  | 10 |  |

**Examples of food items**

| **Food Group** | **Food item** | **Serve size** | **No. of serves/day** | **No. of serves per week** | **Eats or drinks the item less than once a week, or never** |
| --- | --- | --- | --- | --- | --- |
| **Dairy products** | Milk 1% | 1 cup |  |  |  |
|  | Milk 3% |  |  |  |  |
|  | Yogurt, unsweetened |  |  |  |  |
|  | Sour cream | 1 cup |  |  |  |
| **Beef** | Beef steak | 1 serve |  |  |  |
|  | Beef fried patties | 1 serve |  |  |  |
|  | Hamburger | 1 serve |  |  |  |
| **Chicken** | Homemade schnitzel | 1 serve |  |  |  |
|  | Processed schnitzel | 1 serve |  |  |  |
|  | Chicken cooked in water | 1 serve |  |  |  |
| **Vegetables** | Salad | 1 cup |  |  |  |
| **Sweets,**  **Snacks** | Dark chocolate | 4 piece |  |  |  |
| **Drinks** | Cola | 1 cup |  |  |  |

**Exercise questionnaire**

**Please detailed about the following activities: if you are engaged in them, how often, and for how long each session.**

| How long have you continuously engaged in _____? Mention the activity  If more than one activity is mentioned, write in this column the activity which the subject did most often. | What is the duration of the activity each time? | How often do you engage in  _____? Mention the activity. If more than one activity is mentioned, ask about frequency of all the activities together. | Do you regularly engage in ____? Mention the name of the activity. If “yes” continue to Q. 62. If “no” move to next row. Circle the activity selected, if more than one written per row | |
| --- | --- | --- | --- | --- |
| **Aerobic activity** | | | | |
| 1. less than half a year  2. 7 months to 1 year  3. between 1-5 years  4. more than 5 years | ________ min | 1. 4 times a week or more  2. 3 times a week  3. 1-2 times a week  4. 2-3 times a month  5. once a month or less | 1. Yes  2. No- go to next line | Walking outside for fitness propose (medium pace/quick pace) |
| 1. less than half a year  2. 7 months to 1 year  3. between 1-5 years  4. more than 5 years | ________ min | 1. 4 times a week or more  2. 3 times a week  3. 1-2 times a week  4. 2-3 times a month  5. once a month or less | 1. Yes  2. No- go to next line | Walking outside As part of arrangements (walking to work, work, traveling with a cart, etc.) |
| 1. less than half a year  2. 7 months to 1 year  3. between 1-5 years  4. more than 5 years | ________ min | 1. 4 times a week or more  2. 3 times a week  3. 1-2 times a week  4. 2-3 times a month  5. once a month or less | 1. Yes  2. No- go to next line | Walking in the gym at a moderate / fast pace on a treadmill |
| 1. less than half a year  2. 7 months to 1 year  3. between 1-5 years  4. more than 5 years | ________ min | 1. 4 times a week or more  2. 3 times a week  3. 1-2 times a week  4. 2-3 times a month  5. once a month or less | 1. Yes  2. No- go to next line | Running (including on treadmill) |
| 1. less than half a year  2. 7 months to 1 year  3. between 1-5 years  4. more than 5 years | ________ min | 1. 4 times a week or more  2. 3 times a week  3. 1-2 times a week  4. 2-3 times a month  5. once a month or less | 1. Yes  2. No- go to next line | Riding on bike (including in the gym) |
| 1. less than half a year  2. 7 months to 1 year  3. between 1-5 years  4. more than 5 years | ________ min | 1. 4 times a week or more  2. 3 times a week  3. 1-2 times a week  4. 2-3 times a month  5. once a month or less | 1. Yes  2. No- go to next line | Swimming |
| 1. less than half a year  2. 7 months to 1 year  3. between 1-5 years  4. more than 5 years | ________ min | 1. 4 times a week or more  2. 3 times a week  3. 1-2 times a week  4. 2-3 times a month  5. once a month or less | 1. Yes  2. No- go to next line | Aerobic exercise (spinning, kickbox, step) or on a device (exercise bike, ski device, stopper) |
| How long have you continuously engaged in _____? Mention the activity  If more than one activity is mentioned, write in this column the activity which the subject did most often. | What is the duration of the activity each time? | How often do you engage in  _____? Mention the activity. If more than one activity is mentioned, ask about frequency of all the activities together. | Do you regularly engage in ____? Mention the name of the activity. If “yes” continue to Q. 62. If “no” move to next row. Circle the activity selected, if more than one written per row | |
| 1. less than half a year  2. 7 months to 1 year  3. between 1-5 years  4. more than 5 years | ________ min | 1. 4 times a week or more  2. 3 times a week  3. 1-2 times a week  4. 2-3 times a month  5. once a month or less | 1. Yes  2. No- go to next line | Ball games |
| 1. less than half a year  2. 7 months to 1 year  3. between 1-5 years  4. more than 5 years | ________ min | 1. 4 times a week or more  2. 3 times a week  3. 1-2 times a week  4. 2-3 times a month  5. once a month or less | 1. Yes  2. No- go to next line | Dancing, including folk dancing |
| **Resistance activity** | | | | |
| 1. less than half a year  2. 7 months to 1 year  3. between 1-5 years  4. more than 5 years | ________ min | 1. 4 times a week or more  2. 3 times a week  3. 1-2 times a week  4. 2-3 times a month  5. once a month or less | 1. Yes  2. No- go to next line | Exercise for body shaping, including muscle strengthening |
| 1. less than half a year  2. 7 months to 1 year  3. between 1-5 years  4. more than 5 years | ________ min | 1. 4 times a week or more  2. 3 times a week  3. 1-2 times a week  4. 2-3 times a month  5. once a month or less | 1. Yes  2. No- go to next line | Light exercise: yoga, Feldenkrais, Alexander etc. |
| 1. less than half a year  2. 7 months to 1 year  3. between 1-5 years  4. more than 5 years | ________ min | 1. 4 times a week or more  2. 3 times a week  3. 1-2 times a week  4. 2-3 times a month  5. once a month or less | 1. Yes  2. No- go to next line | Other exercise, specify __________ |
